# Screening instruments to detect problematic alcohol use among adults in hospitals and their diagnostic test accuracy: A systematic review

**DOI:** 10.1111/dar.13987

**Published:** 2025-01-19

**Authors:** Jacqueline M. Bisschop, Hendrik J. M. de Jonge, Anja H. Brunsveld‐Reinders, Dike H. van de Mheen, Jolanda J. P. Mathijssen, Andrea D. Rozema

**Affiliations:** ^1^ Jeroen Bosch Hospital s‐Hertogenbosch the Netherlands; ^2^ Department Tranzo Tilburg School of Social and Behavioural Sciences Tilburg the Netherlands; ^3^ Epidemiology and Data Science Amsterdam University Medical Center, University of Amsterdam Amsterdam the Netherlands; ^4^ Department Quality and Patient Safety Leiden University Medical Center Leiden the Netherlands

**Keywords:** adults, alcohol use disorder, hospital, problematic alcohol use, screening instrument

## Abstract

**Issues:**

Alcohol consumption can cause physical, psychological and social problems that can result in hospitalisations. Screening in hospitals helps to determine which patients should be given interventions. In this review, we aimed to summarise the diagnostic test accuracy (DTA) of screening instruments for problematic alcohol use among adults in hospitals.

**Approach:**

We searched three databases for studies that assessed validated screening instruments for problematic alcohol use among hospitalised adults. We used the Quality Assessment of Diagnostic Accuracy Studies 2 tool to evaluate the risk of bias.

**Key Findings:**

We included 20 studies in the review. In 11 studies performed in the emergency departments, 5 instruments had a high DTA: the AUDIT, the AUDIT‐C, the RAPS4‐QF, the 2‐question screener and HOLD 5. In the eight studies with inpatients, three instruments had a high DTA: the AUDIT, CAGE‐C and CAGE +10 items. Finally, only one of the included studies evaluated outpatients; the authors reported a high DTA for the AUDIT‐C.

**Implication and Conclusions:**

The results indicate that the AUDIT is an appropriate screening instrument for both inpatients and patients in the emergency department. Moreover, for patients in the emergency department, the AUDIT‐C and the RAPS4‐QF can be used. More research is needed on outpatients, and several screening instruments have only been validated with one study (i.e., the 2‐question screener, DSM‐IV‐2, HOLD 5, CAGE‐C and CAGE +10 questions).

**Key Points**

- We provide an overview of the sensitivity and specificity of alcohol screening instruments in hospitalised adults.
- We used the QUADAS‐2 tool to assess the risk of bias.
- Screening instruments are the best way to detect problematic alcohol use.
- The studies included 22 different screening instruments, 12 descriptions and 6 reference standards.
- The Alcohol Use Disorder Identification Test is an appropriate screening instrument for both inpatients and patients in the emergency department. Moreover, for patients in the emergency department, Alcohol Use Disorder Identification Test for Consumption and the Rapid Alcohol Problems Screen 4 + quantity and frequency question can be used.

## 1 INTRODUCTION

Alcohol consumption is a global, multi‐layered problem that can cause physical, psychological and social problems, which may result in hospitalisations and increased health‐care costs [1, 2]. In 2020, 1.34 billion people throughout the world consumed harmful amounts of alcohol [3]. In Europe, there were 71,400 deaths in 2016 attributable to alcohol use disorder (AUD) [1, 2]. This medical condition is characterised by an impaired ability to stop or control alcohol use despite adverse social, occupational or health consequences [4].

Globally, in hospitals, one‐sixth of the (non‐psychiatric) patients have AUD [5, 6]. Moreover, AUD is related to over 200 diseases [2], including various forms of cancer, dementia and injuries [7]. By hospitals, we mean an institution that is built, staffed and equipped for the diagnosis of disease; for treatment, both medical and surgical, of the sick and the injured; and for their housing during this process [8]. To help patients reduce their alcohol consumption and to prevent the onset and progression of AUD, it is recommended to use screening, brief intervention and referral to treatment in hospitals [9, 10, 11]. In this systematic review, we use the term ‘problematic alcohol use’ to refer to all aspects of inappropriate alcohol use (AUD, alcohol problem, alcohol abuse, hazardous drinking, harmful drinking, alcohol dependence, etc.).

Of note, only a minority of hospitals screen for problematic alcohol use due to the lack of a clear protocol, training or collaboration with other (external) partners in health care [12]. Therefore, most patients with problematic alcohol use remain undetected and receive no alcohol treatment [13, 14, 15]. Indeed, alcohol interventions in hospitals still seem to be in their infancy. Researchers have recommended all hospitals to implement screening for problematic alcohol use and appropriate interventions [12].

The authors of several studies have evaluated different screening instruments for problematic alcohol use detection in hospitals. In 2015, researchers undertook a cross‐sectional study focussing on screening instruments for substance use (including alcohol) in a geriatric hospital and the community health setting [16]. They recommended routine screening for alcohol and substance use and found that the Alcohol Use Disorder Identification Test for Consumption short form (AUDIT‐C; see Table A1 for a full description and additional information of all the screening instruments discussed in this review) with a cutoff of ≥5 was optimal for detecting risky alcohol use, compared with the Alcohol Use Disorder Identification Test (AUDIT), the Alcohol, Smoking and Substance Involvement Screening Test, and the Cutting Down, Annoyance by Criticism, Guilty Feeling, Eye‐Openers (CAGE) questionnaires. However, the authors performed this study in a geriatric hospital, and therefore they could not draw conclusions regarding the general adult patient population of hospitals. Additionally, Verhalle et al. [17] investigated how to screen for at‐risk alcohol use in transplant patients; they concluded that the AUDIT‐C might be a suitable screening instrument. Again, they performed their study with a specific adult population (i.e., transplant patients). Moreover, in 2011 a systematic review was performed to assess the alcohol screening tools in the emergency department (ED) [18]. The authors recommended the Functional Analysis Screening Tool (FAST) and the Paddington Alcohol Test (PAT) as screening instruments.

To decide which screening instrument to use for problematic alcohol use, it is important for clinicians to know the advantages and disadvantages of the different instruments. However, there is no recent systematic overview of which screening instruments are best for the general adult population in hospitals. Most hospitals treat patients with problematic alcohol use when patients are in the ED (including both inpatients and outpatients), when they are hospitalised (i.e., inpatients), or when they do not stay the night after treatment/consultation (i.e., outpatients). In this review, we aimed to summarise the diagnostic test accuracy (DTA) of screening instruments for problematic alcohol use among adults in hospitals.

## 2 METHOD

### 2.1 Search strategy

To identify relevant articles, we searched the PubMed, PsycINFO and Embase databases. We selected these databases to fully represent medical, psychological and pharmacological articles, respectively. Table A2 presents the complete search strings, including MeSH terms and keywords. We registered the protocol of this systematic review with the International Prospective Register of Systematic Reviews (PROSPERO), under number CRD42022291306 [19].

### 2.2 Inclusion and exclusion criteria

We included peer‐reviewed empirical studies that were written in English; published from 2001 to 2021; had a quantitative design; used validated screening instruments (i.e., instruments that are used according to standardised protocols developed from previous studies in different populations) for problematic alcohol use among adults (>18 years) specifically in hospitals; and included measurements of their DTA, reporting at a minimum the sensitivity and specificity (and, ideally, the positive predictive and negative predictive values). We excluded studies with another dependent variable (e.g., alcohol withdrawal scales, biomaterial scales, craving scales and intoxication scales); that tested translations of existing validated screening instruments (because these studies mainly focussed on the accuracy of the translation and not on the screening instrument itself); and that reported outcomes (i.e., sensitivity and specificity) for males and females separately only, because we aimed to find the most adequate predictive screener for detecting problematic alcohol use among *adults in general*.

### 2.3 Selection of the studies

**FIGURE 1:**
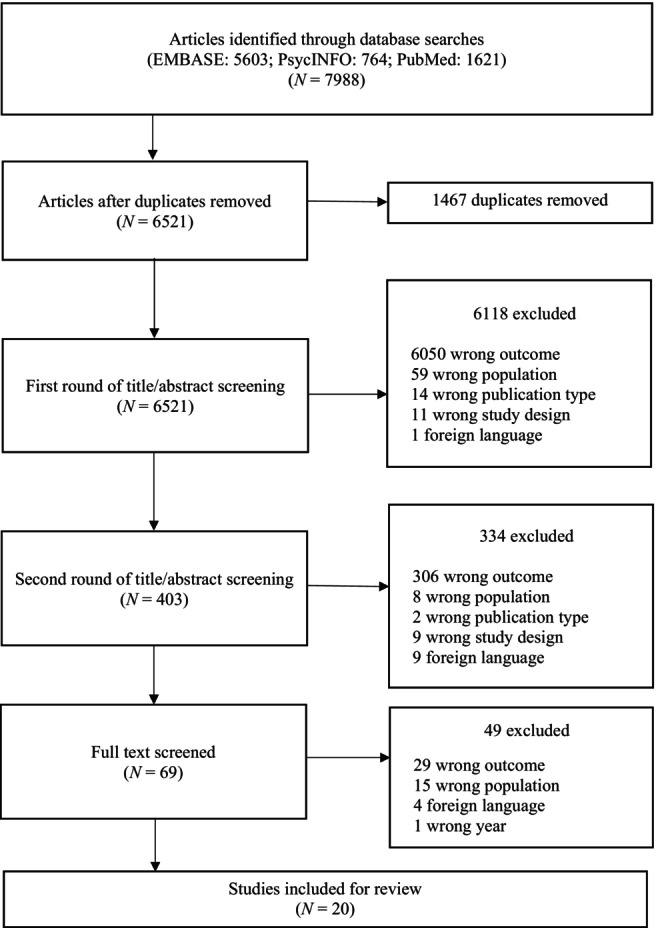
Preferred reporting items for systematic reviews and meta‐analyses flow diagram.

This systemic review is reported in line with the Preferred Reporting Items for Systematic Reviews and Meta‐Analyses (PRISMA) guidelines [20] and the guidelines for DTA [21]. Figure 1 presented the PRISMA flow diagram. The studies were selected using Rayyan and Covidence, software that facilitates the screening process for systematic reviews [22, 23]. First, three authors (Jacqueline M. Bisschop, Hendrik J. M. de Jonge and Andrea D. Rozema) were involved in the screening process. The first author (Jacqueline M. Bisschop) screened 100% of the titles and abstracts, and the other two authors, Hendrik J. M. de Jonge and Andrea D. Rozema, screened 20% of the titles and abstracts. Inconsistencies were discussed with all three authors and there had to be a minimal agreement of >95% in the screening between Jacqueline M. Bisschop and the other two authors to approve this procedure. This was performed by separately screening 20% of the titles and abstracts and comparing the differences. The minimal agreement of >95% was achieved. Second, all full articles were read independently by at least two authors: Jacqueline M. Bisschop read 100%, and Hendrik J. M. de Jonge and Andrea D. Rozema each read 50%. This process left a subset of relevant studies for inclusion. In the case of disagreement about study eligibility or a lack of consensus between two reviewers, the third reviewer was consulted, and disagreements were discussed until a consensus was reached. To ensure that no articles were missed, a backward reference check was executed (i.e., checking the reference lists from each included study references for missing articles).

### 2.4 Data extraction

Two authors (Jacqueline M. Bisschop 100%, and Hendrik J. M. de Jonge and Andrea D. Rozema, 50% each) independently extracted the following data for each included study: author, year, country, mean age, gender, the severity of the alcohol problems (i.e., different descriptions of problematic alcohol use), and the reference standard (Table 1), and independently performed a quality assessment of the included articles using the Quality Assessment of Diagnostic Accuracy Studies 2 (QUADAS‐2) [24]. The QUADAS‐2 tool is recommended in the DTA guideline when performing a systematic review to evaluate the risk of bias and the applicability of primary diagnostic accuracy studies. This instrument consists of four key domains, namely: (i) patient selection; (ii) the index test; (iii) the reference standard; and (iv) flow and timing.

**TABLE 1:** Characteristics of the included studies.

Author/s (year), country [reference]	*N*	Mean age, years	Gender	Population	Severity of problematic alcohol use	Reference standard
Shenvi et al. (2020), USA [27]	222	73	122 (55%) female	ED	98 (44%) high‐risk drinkers	Timeline follow back
			100 (45%) male			
Larsson and Nehlin (2016), Sweden [28]	898	49.2 (SD 17.3) Female	466 (52%) Female	Outpatients	102 (11.4%) Hazardous or harmful use	AUDIT
		52.2 (SD 17.3) Male	432 (48%) Male		10 (1.1%) Heavy abuse	
					8 (0.9%) Dependence	
					120 (13.4%) Above hazardous level	
Fulbrook et al. (2015), Australia [29]	637	49.9 (SD 20.0)	321 (50.4%) Female	ED	AUDIT risk zone scores	AUDIT
			316 (49.6%) Male		0–7: 488 (76.6%)	
					8–15: 104 (16.3%)	
					16–19: 17 (2.7%)	
					>20: 28 (4.4%)	
Geneste et al (2012)., France [30]	164	45.7 (SD 11.6)	122 (74.39%) Male	ED	19 (3%) Alcohol abuse	DSM‐IV + MINI
			42 (25.61) Female	Only patients with AAI	128 (78%) Alcohol dependence	
Richoux et al (2011)., France [31]	1079	46.6 (SD 20)	586 (54%) Female	ED	103 (9.5%) Alcohol abuse/dependence	DSM‐IV + MINI
			493 (46%) Male			
Okay et al. {2010), Turkey [32]	800	45.59 (SD 13.28)	406 (50.8%) Female	Inpatients	60 (7.5%) Alcohol addiction	DSM‐IV (SCID‐I)
			394 (49.3%) Male		11 (1.4%) Alcohol abuse	
Kelly et al. (2009), USA [33]	181	Range 18–20.9	104 (57%) Male	ED	65 (36%) Alcohol abuse/dependence	DSM‐IV
			77 (43%) Female	Adolescents		
Cremonte and Cherpitel (2008), Argentina [34]	643	36	405 (63%) Male	ED	58 (9%) DSM‐IV abuse	ICD‐10 and DSM‐IV
			238 (37%) Female	Current drinkers	58 (9%) DSM dependence	
					58 (9%) ICD‐10 harmful drinking	
					58 (9%) ICD‐10 dependence	
Bradshaw et al. (2008), Ireland [35]	210	42.1 (SD 14.3)	121 (58%) Male	Inpatients	46 (22%) Drink in excess	DSM‐IV + AUDIT
			89 (42%) Female	Data from 2004 to 2005	19 (9%) Alcohol use disorder	
Zierau et al. (2005), Denmark [36]	130	50 (range 18–82)	75 (58%) Female	Inpatients	33 (25%) Alcohol problem	DSM‐III + ICD‐10
			55 (42%) Male	Orthopaedic surgery		
Chen et al. (2005), Taiwan [37]	422	41.9 (SD 12.9) Male	268 (63.5%) Male	Inpatients	59 (14%) Alcohol use disorder	DSM‐IV
		43.7 (SD 13.9) Female	154 (36.5%) Female		14 (3%) Alcohol abuse	
					45 (11%) Alcohol dependence	
Castells and Furlanetto (2005), Brazil [38]	729	50 (SD 17)	486 (66.7%) Male	Inpatients	48 (6.6%) Alcohol‐dependent patients	DSM‐IV + MINI
			243 (33.3%) Female			
Kelly et al. (2004), USA [39]	93	19 (SD 0.9)	51 (55%) Male	ED	38 (41%) Alcohol use disorder	DSM‐IV
		Range 18–20	42 (45%) Female	Adolescents	25 (27%) Alcohol abuse	
					13 (14%) Alcohol dependence	
Cherpitel and Bazargan (2003), USA [40]	395	African American	African Americans 130 (68%) Male	ED	African American	DSM‐IV + CIDI
		32% 18–29	61 (32%) Female		44 (23%) Alcohol dependence	
		48% 30–49			54 (28%) Alcohol abuse	
		21% ≥ 50				
			Hispanic 204			
		Hispanic	102 (50%) Male			
		41% 18–29	102 (50%) Female		Hispanic	
		35% 30–49			31 (15%) Alcohol dependence	
		24% ≥ 50			43 (21%) Alcohol Abuse	
Hodgson et al. (2002), UK [41]	666 ED	76% ≥ 25^d^	53% Male	ED	N unclear Hazardous drinkers	AUDIT ≥8
			47% Female			
Hearne et al. (2002), Ireland [15]	759		381 (50%) Female	Inpatients	37 (5%) Alcohol abuse	DSM‐IV (subset only)
			378 (50%) Male	Data from 1999	42 (6%) Alcohol dependence	
Aertgeerts et al. (2002), Belgium [42]	233	62 (interquartile range: 19–74)	233 (100%) Male	Inpatients	29 (12.4%) Alcohol abuse/dependence	DSM‐III‐R + CIDI
					10 (4.2%) Alcohol abuse	
					19 (8.2%) Dependence	
Gomez et al. (2001)Spain (Gran Canaria) [43]	179	49 (SD 12–15)	Of 99 inpatients with ARP:	Inpatients	99 (100% ARP group) Hazardous drinkers	ICD‐10
			82 (83%) Male, 17 (17%) Female	Data 1997–1998		
			Control group 80 patients:			
			64 (80%) Male, 16 (20%) Female			
Cherpitel (2001), USA [44]	ED 1091	ED	ED 415 (38%) Male, 677 (62%) Female	ED	98 (9%) Alcohol dependence	DSM‐IV + ICD‐10
		47% < 30				
		38% 30–49				
		16% ≥ 50				
Borges and Cherpitel (2001), USA and Mexico [45]	869	<30 (41%)	626 (72%) Male	ED	243 (28%) ADAAHM	DSM‐IV + ICD‐10
		>30 (59%)	243 (28%) Female	Data from 1995 to 1996	130 (15%) ADS	
				Only current drinkers		

Abbreviations: AAI, acute alcohol intoxication; AUDIT, Alcohol Use Disorder Identification Test; DSM‐IV, Diagnostic and Statistical Manual of Mental Disorders fourth version; ED, emergency department; ICD‐10, International Statistical Classification of Diseases and Related Health Problems 10th Revision; SCID‐I, Structured Clinical Interview For DSM‐IV.

### 2.5 Analyses

The DTA of the studies included the sensitivity and specificity, and, if possible, the positive and negative predictive values (Tables 2 and 3). See Table A2 for the full outcome table of all studies. After inclusion and data extraction, we assessed whether a meta‐analysis was feasible, based on the homogeneity of the study designs, the consistency of the outcome measures, sufficient data reporting, and the methodological quality of the studies [21]. If a meta‐analysis was not feasible, then we followed the synthesis without meta‐analysis reporting guidelines [25].

**TABLE 2:** Full description of the screening instruments.

Abbreviation	Full description	Number of questions	Additional information
AUDIT	Alcohol Use Disorder Identification Test	10	Original
AUDIT‐C	Alcohol Use Disorder Identification Test for consumption short form	3	Original short form (first 3 items from the AUDIT)
AUDIT‐PC	Alcohol Use Disorder Identification Test Primary Care	5	Short form (5 items from the AUDIT)
AUDIT‐3	Single item 3 from the AUDIT Use Disorder Identification Test	1	Single question (item 3 from AUDIT)
CAGE	C—Cutting Down	4	Original
	A—Annoyance by Criticism		
	G—Guilty Feeling		
	E—Eye‐Openers		
CAGE‐C	C—Cutting Down	6	CAGE +2/3 additional questions
	A—Annoyance by Criticism		
	G—Guilty Feeling		
	E—Eye‐Openers		
	Copenhagen		
CAGE+10	C—Cutting Down	14	CAGE +10 additional questions
	A—Annoyance by Criticism		
	G—Guilty Feeling		
	E—Eye‐Openers		
	+ 10 additional questions		
CRAFFT	Car, Relax, Alone, Forget, Friends, Trouble	6	Original for substance use in adolescents
FAST	Functional Analysis Screening Tool	4	Short form (4 items from the AUDIT)
MAST	Michigan Alcohol Screening Test	25	Original
TWEAK	T—Tolerance	5	Original for pregnant women
	W—Worried		
	E—Eye Opener		
	A—Amnesia		
	K—Cut Down		
RAPS	Rapid Alcohol Problems Screen	5	Combination of items from CAGE, TWEAK, the MAST, and the AUDIT
RAPS‐4	Rapid Alcohol Problems Screen 4 questions	4	Short form (4 items from the RAPS)
RAPS‐QF	Rapid Alcohol Problems Screen 4 + quantity and frequency question	6	Short form (4 items from the RAPS) + 2 additional questions
BMAST	The Brief Michigan Alcohol Screening Test	10	Short form (10 items from the MAST)
Fiveshot	Five shot questionnaire	5	Combination of items from the AUDIT and CAGE
HOLD 5	Being able to hold at least 5 drinks	1	Single question
5 Monthly	Drinking ≥5 drinks at one time at least monthly	1	Single question
DSM‐IV‐2	2 items from the DSM‐IV	2	2 items from the DSM‐IV
WCQ	Weekly consumption question	1	Single question
HED	Heavy episodic drinking	1	Single question
2‐question screener	2‐question screener	2	Original
ASSIST	Alcohol, Smoking and Substance Involvement Screening Test	8	Original, all substance use
PAT	Paddington Alcohol Test	5	Original for the emergency department

Abbreviation: DSM‐IV, Diagnostic and Statistical Manual of Mental Disorders, fourth version.

**TABLE 3:** Overview of the diagnostic test accuracy of the included screening instruments used in different populations in hospitals^a^ ^,^ ^b^.

Screening instrument	Sensitivity	Specificity	Patient category	Description of problematic alcohol use	Specific populations
AUDIT (without cutoff)	0.65–0.93	0.77–0.95	ED [31, 34^c^, 40, 44]	High‐risk drinkers [27]	Older adults^c^ [27]
AUDIT ≥4	**0.93**	**0.78** ^d^	ED^c^ [27]	Alcohol abuse [15, 30, 31, 34, 39, 42]	Acute alcohol intoxication [30]
AUDIT ≥5	**0.83**	**0.85**	Inpatients [42]	Alcohol dependence [15, 30, 31, 34, 39, 42, 44]	Only current drinkers [34]
AUDIT ≥8	0.75–0.97	0.84–0.91	Inpatients [15, 37]	Harmful drinking [34]	Adolescents [39]
AUDIT ≥10	0.75–0.82	0.78–0.97	ED^c^ [39], inpatients [15]	Alcohol use disorder [37, 39]	
AUDIT ≥12**	0.60–0.88	0.88–0.98	ED^c^ [30], inpatients [15]		
AUDIT ≥18***	**0.8**	**0.83**	ED^c^ [30]		
AUDIT‐C (without cutoff)	**0.85**	**0.88**	Outpatients [28]	Hazardous or harmful use [28]	Adolescents [33]
AUDIT‐C ≥ 5	0.69	0.87	Inpatients [42]	Heavy abuse [28]	
AUDIT‐C ≥ 6	0.74	0.77	ED^c^ [33]	Dependence [28, 33, 42]	
				Above hazardous level [28]	
				Alcohol abuse [33, 42]	
RAPS4 (without cutoff)	0.74–0.97	0.79–0.93	ED [44], ED^c^ [45], ED [40]	Alcohol abuse [30, 33, 34, 39, 45]	Acute alcohol intoxication [30]
RAPS4‐QF (without cutoff)	**0.86–0.98**	**0.83–0.87**	ED [34, 40]	Alcohol dependence [30, 34, 39, 44, 45]	Adolescents [33, 39]
RAPS4 ≥ 1**	0.95	0.65	ED^c^ [30]	Alcohol use disorder [39]	Only current drinkers [34]
RAPS4 ≥ 2***	0.84	0.64	ED^c^ [30]	Harmful drinking [34]	
RAPS4‐QF ≥3	0.79–0.92	0.53–0.77	ED^c^ [30, 33, 39], ED^c^ [34]		
FAST ≥3	0.83–1.0	0.73–0.94	ED^c^ [33], inpatients [35], ED [41]	Alcohol abuse/dependence [33]	Adolescents [33]
				Drink in excess [35]	
				Alcohol use disorder [35]	
				Hazardous drinkers [41]	
CRAFFT ≥3	0.69–0.82	0.67–0.73	ED^c^ [33^c^, 39]	Alcohol abuse [33, 39]	Adolescents [33, 39]
				Dependence [33, 39]	
				Alcohol use disorder [39]	
TWEAK	0.66–0.98	0.65–0.92	ED [34^c^, 44]	Alcohol abuse [34]	Only current drinkers [34]
				Dependence [34, 44]	
				Harmful drinking [34]	
CAGE (without cutoff)	0.48–0.89	0.58–0.94	ED^c^ [39], ED [34^c^, 44], inpatients [43]	High‐risk drinkers [27]	Older adults^c^ [27]
CAGE ≥1	0.72–0.75	0.85–0.92	ED [31, 34^c^, 42]	Alcohol abuse [15, 30, 31, 34, 39, 42]	Acute alcohol intoxication [30]
CAGE ≥2**	0.45–0.94	0.65–1.0	Inpatients [15], ED^c^ [27, 30], ED^c^ [30]	Alcohol dependence [15, 30, 31, 34, 39, 42]	Only current drinkers [34]
CAGE ≥3***	0.89	0.61		Harmful drinking [34]	Adolescents [39]
				Alcohol use disorder [39]	
				Hazardous drinkers [43]	
BMAST ≥5	0.04–0.37	0.95–1.0	Inpatients [15], ED [34^c^, 44]	Alcohol abuse [15, 34]	Only current drinkers [34]
				Dependence [15, 34, 44]	
				Harmful drinking [34]	
2‐question screener	**0.98**	**0.87**	ED^c^ [27]	High‐risk drinkers [27]	Older adults^c^ [27]
CAGE‐C	**0.94**	**0.88**	Inpatients [36]	Alcohol problem [36]	
CAGE +10 items	**0.94**	**0.86**	Inpatients [38]	Alcohol‐dependent patients [38]	
DSM‐IV‐2	**0.88**	**0.90**	ED^c^ [33]	Alcohol abuse/dependence [33]	Adolescents [33]
HOLD ≥5	**0.83**	**0.82**	ED [44]	Alcohol dependence [44]	
Fiveshot ≥2.5	**0.79** ^d^	**0.88**	Inpatients [42]	Alcohol abuse dependence [42]	
MAST	0.74	0.98	Inpatients [32]	Alcohol addiction [32]	
				Alcohol abuse [32]	
5 MONTHLY	0.74	0.92	ED [44]	Alcohol dependence [44]	
AUS PAT	0.73	0.91	ED [29]	AUDIT risk zone scores [29]	
RUFT‐Cut ≥3	0.80	0.68	ED^c^ [33]	Alcohol abuse/dependence [33]	Adolescents (33)
AUDIT‐PC	0.69	0.91	Inpatients [42]	Alcohol abuse/dependence [42]	
HED	0.69	0.91	Outpatients [28]	Hazardous or harmful use [28]	
				Heavy abuse [28]	
				Dependence [28]	
				Above hazardous level [28]	
AUDIT‐item 3	0.55	0.96	Outpatients [28]	Hazardous or harmful use [28]	
				Heavy abuse [28]	
				Dependence [28]	
				Above hazardous level [28]	
WCQ	0.12	0.91	Outpatients [28]	Hazardous or harmful use [28]	
				Heavy abuse [28]	
				Dependence [28]	
				Above hazardous level [28]	

Abbreviations: AUDIT, Alcohol Use Disorder Identification Test; AUDIT‐C, Alcohol Use Disorder Identification Test for consumption short form; BMAST, Brief Michigan Alcohol Screening Test; ED, emergency department; FAST, Functional Analysis Screening Tool; HED, heavy episodic drinking; MAST, Michigan Alcohol Screening Test; PAT, Paddington Alcohol Test; RAPS‐4, Rapid Alcohol Problems Screen 4 questions; RAPS‐QF, Rapid Alcohol Problems Screen 4 + quantity and frequency question; TWEAK, Tolerance, Worried, Eye opener, Amnesia, Cut down; WCQ, weekly consumption question.

^a^ Bold numbers are sensitivity and specificity combinations >0.78 (i.e., the highest combinations).

^b^ The positive predictive and negative predictive values for specific studies are presented in Table A2.

^c^ Specific populations.

^d^ Included in the high sensitivity and specificity combinations due to the closeness to the >0.8 cutoff.

### 2.6 Risk of bias assessment

We assessed the risk of bias by using the QUADAS‐2 risk of bias tool, as described in Section 2.4. The outcomes of the QUADAS‐2 assessment are summarised in Table 4 and Section 3.5.

**TABLE 4:** The quality assessment of diagnostic accuracy studies 2 tool risk of bias assessment.

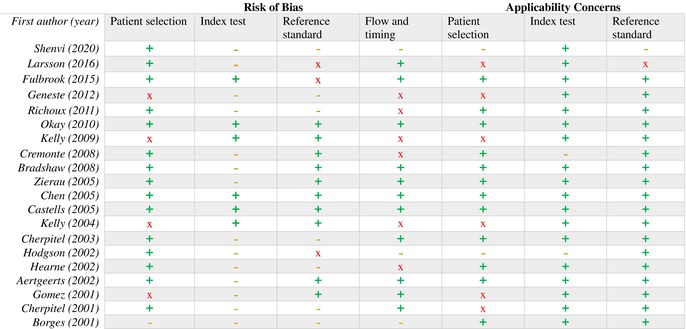

*Note*: The colors indicate the risk of bias: “green/+” for low, “yellow/‐” for unclear, and “red/x” for high.

## 3 RESULTS

### 3.1 Study selection

In the initial search, we found 7988 articles. After removing duplicates using the duplication guideline [26], 6521 articles remained. After screening the titles and abstracts, 69 articles remained for full‐text screening. Finally, we included 20 studies for data extraction and quality assessment [15, 27, 28, 29, 30, 31, 32, 33, 34, 35, 36, 37, 38, 39, 40, 41, 42, 43, 44, 45]. We did not find additional relevant studies when we searched the reference lists of the 20 included studies.

All of the included studies had a cross‐sectional design; however, due to the clinical heterogeneity—that is, differences in screening instruments, reference standards, populations and descriptions of problematic alcohol use—a meta‐analysis was impossible [21]. Therefore, we followed the synthesis without meta‐analysis guidelines to generate a narrative synthesis instead [25]. We grouped the studies for synthesis according to the DTA of the included screening instruments, categorising the highest to lowest sensitivity and specificity combinations (Table 3). Table 4 shows the results of the QUADAS‐2 risk of bias tool, with outcomes in three categories: low risk of bias (green dots), unclear risk of bias (yellow dots) and high risk of bias (red dots). In the following subsections, we describe the differences in study characteristics, the DTA of the screening instruments, the use of screening instruments versus recognition by staff or biological markers, and the quality of the included studies.

### 3.2 Study characteristics

Ten of the studies were performed in Europe [15, 28, 30, 31, 32, 35, 36, 41, 42, 43] and 10 were performed on other continents (North America, Australia, South America and Asia) [27, 29, 33, 34, 37, 38, 39, 40, 44, 45]. The sample sizes ranged from 93 patients [39] to 1079 patients [31]. There were differences in the hospital departments in which the studies were performed. Specifically, 11 studies were conducted in the ED [27, 29, 30, 31, 33, 34, 39, 40, 41, 44, 45], 8 studies involved inpatients (i.e., on clinical wards) [15, 32, 35, 36, 37, 38, 42, 43], and 1 study involved outpatients [28]. The included studies used 12 different descriptions of alcohol problems: high‐risk drinkers [27], hazardous or harmful use [28, 41, 43], heavy abuse [28], dependence [15, 28, 30, 31, 33, 34, 37, 38, 39, 40, 42, 44, 45], above the hazardous level [28], AUDIT risk zones [29], alcohol abuse [15, 30, 31, 32, 33, 34, 37, 39, 40, 42, 45], alcohol addiction [32], harmful drinking [34, 45], AUD [35, 37, 39], drink in excess [35] and alcohol problems [36]. There were seven different (combinations of) reference standards: Diagnostic and Statistical Manual of Mental Disorders fourth version (DSM‐IV) [15, 32, 33, 35, 37, 39], DSM‐IV + International Statistical Classification of Diseases and Related Health Problems 10th Revision (ICD‐10) [34, 40, 44, 45], DSM‐IV + The Mini‐International Neuropsychiatric Interview [30, 31, 38], DSM‐III + ICD‐10 [36, 42], ICD‐10 [43], the AUDIT [28, 29, 41] and timeline follow back [27]. Lastly, there were many specific populations described. For example, patients that were ≥65 years old [27], patients with acute alcohol intoxication (AAI) [30], adolescents aged 18–21 years old [33, 39] and current drinkers [34, 45].

### 3.3 Diagnostic test accuracy of the screening instruments

Twenty‐two different screening instruments were described in the 20 studies (the AUDIT, the AUDIT‐C, the AUDIT‐PC, the AUDIT‐3, CAGE, CAGE‐C, CAGE +10 questions, CRAFFT, the FAST, the MAST, the TWEAK, the RAPS, the RAPS‐4), the RAPS‐QF, the BMAST, Fiveshot, HOLD 5, 5 Monthly, DSM‐IV‐2, the WCQ, the HED and the two‐question screener. The studies evaluated up to seven screening instruments. See Table 2 for the full names and descriptions of the screening instruments.

Table 3 shows the best cutoff points that were recommended by the authors of each study. Only 7 of the 20 included studies mentioned the positive and negative predictive values [15, 36, 37, 38, 39, 42, 43]. It was not possible to calculate the missing predictive values [46] because of multiple different descriptions of prevalence. See Table A1 for the full outcome table of all the studies (including the positive and negative predictive values when mentioned in the specific studies).

We chose a cutoff point of >0.8 for both sensitivity and specificity; it corresponds to the cutoff points recommended by the authors of the included articles (i.e., the most adequate screening instruments). For some of the screening instruments, different cutoff values were used for descriptions/severity of problematic alcohol use in the different studies (e.g., using the AUDIT, ≥8 to detect harmful drinking and ≥15 to detect moderate to severe AUD). Table 3 provides the full details regarding the DTA and additional information (e.g., the description of problematic alcohol use). We marked the most adequate screening instruments in bold. Below, we emphasise the highest and lowest DTA combinations.

For patients in the ED, there was high DTA for five screening instruments: the AUDIT with a cutoff of ≥4 [27], the AUDIT with a cutoff of ≥18 [30], the RAPS4‐QF [34, 40], the two‐question screener [27], DSM‐IV‐2 [33] and HOLD 5 [44]. This was investigated among a group of patients with an extremely high percentage of alcohol problems [27, 30, 33], older adults [27], current drinkers [34], patients with AAI [30], adolescents [33] and regular patients [40].

For inpatients, there was a high DTA for three screening instruments: the AUDIT with a cutoff of ≥5 [42], CAGE‐C [36] and CAGE +10 questions [38]. This was investigated among a group of regular patients [36, 38, 42].

For outpatients, there was high DTA for one screening instrument, namely the AUDIT‐C [28]. This was investigated among regular patients [28].

There was low DTA for three screening instruments: AUDIT‐item 3 [28], the WCQ [28] and the BMAST [15, 34, 44]. This was investigated among outpatients [28], inpatients [15] and patients in the ED [34, 44], with the patients representing hospitalised adults [15, 28, 44] or only current drinkers [34].

Five of the high‐scoring screening instruments were only reviewed in one study—that is, the two‐question screener [27], CAGE‐C [36], CAGE +10 questions [38], DSM‐IV‐2 [33] and HOLD 5 [44]. The DTA of CAGE showed inconsistencies across the included studies [15, 27, 30, 31, 34, 39, 42, 43, 44].

### 3.4 The use of screening instruments versus recognition by staff or biological markers

Four studies compared ‘recognition by staff of the alcohol problem’ and biological markers with the screening instruments. One study compared the recognition by staff of an alcohol problem (i.e., professional assessment without a screening instrument), resulting in 18% recognition. The authors compared the DTA of this professional assessment with the DTA of the AUDIT, CAGE and the BMAST, and found the screening instruments had a higher DTA [15]. Three studies compared biological markers, namely carbohydrate‐deficient transferrin, with a sensitivity range of 10%–70% and a specificity range of 75%–96% [36, 42, 43], and γ‐glutamyl transferase [42, 43] with CAGE [36, 43] and the AUDIT [42], the screening instruments had a higher DTA.

### 3.5 Quality of the included studies

Most domains regarding patient selection, applicability of the index test, and the reference standard had a low risk of bias and applicability (Table 4). There were five reasons for unclear results or a high risk of bias and applicability. First, the most unclear results were based on the question ‘Were the index test results interpreted without knowledge of the results of the reference standard?’, because this was rarely mentioned in the included studies. Second, the AUDIT was used as a reference standard in three studies [28, 29, 41]. Third, the patient selection in six studies concerned a specific population and therefore did not completely represent all hospitalised adults. Fourth, the flow and timing of the screening instruments (e.g., which order or was there enough time between the tests) were often poorly mentioned, or there were concerns about risk of bias.

## 4 DISCUSSION

The objective of this review was to summarise the DTA of screening instruments for problematic alcohol use among hospitalised adults. We found 20 articles, mostly about patients in the ED, followed by inpatients and outpatients. The authors of these 20 articles evaluated 22 different screening instruments. Furthermore, 12 different descriptions of problematic alcohol use and 6 different reference standards were used. Due to these differences, a meta‐analysis was not possible. However, it was possible to generate an overview of the screening instruments, describing the sensitivity and specificity cutoffs recommended by the studies, the description of problematic alcohol use, and specific populations (e.g., adolescents or patients with AAI). This overview provides comprehensive and practical insight into the available screening instruments and their DTA. Additionally, it summarises the variations in studies conducted within hospital settings regarding screening instruments for detecting problematic alcohol use.

It is important to note that there are several screening instruments with a high DTA. Focussing on the most adequate predictive screening instruments, we identified the AUDIT as an adequate screening instrument for inpatients as well as patients in the ED. The instruments with the highest sensitivity and specificity combinations for outpatients in the ED were the AUDIT [27, 30], the RAPS4‐QF [34, 40], the two‐question screener [27], DSM‐IV‐2 [33], and HOLD 5 [44]. Moreover, the AUDIT‐C [28] can also be used for outpatients. The instruments with the highest sensitivity and specificity combinations for inpatients were the AUDIT [42], CAGE‐C including two additional questions [36] and CAGE +10 questions [38]. Furthermore, the DTA of the AUDIT, the AUDIT‐C and the RAPS4‐QF was often reviewed in multiple studies among different hospital populations. They showed consistently high sensitivities and specificities across the included studies. Caution, however, is advised when using the two‐question screener, DSM‐IV‐2, and HOLD 5 with patients in the ED, and CAGE‐C including two additional questions and CAGE +10 questions with inpatients, as the sensitivity and specificity of these screening instruments have only been demonstrated in a single study each. More research is needed on these instruments in different hospital settings and with different patients before recommendations about their use in hospital settings can be made. Furthermore, the number of studies focussing on outpatients was small [28]. This represents a potential direction for further research. Although not the primary focus of our study, it looks like biological markers and recognition by staff are not recommended over screening instruments due to very low sensitivity and specificity scores.

In their 2011 systematic review, Jones et al. [18] recommended the FAST and the Paddington Alcohol Test as screening instruments for patients in the ED, a finding that is contrary to our results. However, those authors focussed on screening instruments for patients in the ED and did not include all screening instruments such as the AUDIT‐C. Of note, in 2011, the AUDIT‐C had not been sufficiently studied for patients in the ED.

In their 2009 review, Meneses‐Gaya et al. [47] investigated the psychometric properties of the AUDIT, comparing primary care patients, psychiatric patients and hospitalised patients (i.e., comparing mostly different settings and a few different screening instruments). Consistent with our findings, those authors confirmed that the AUDIT and its short versions (e.g., the AUDIT‐C) present excellent sensitivity and specificity, often superior to other tests, in various populations [47]. Moreover, the authors stated that the original CAGE (with only four questions) had the lowest sensitivity and specificity and a limited ability to detect AUD. However, due to its simple administration, CAGE continues to be widely used all over the world. In the present review, CAGE (without additional questions) also showed inconsistencies across studies, with a sensitivity of 45%–94% and a specificity of 58%–100% [15, 27, 30, 31, 34, 39, 42, 43, 44]. One study indicated that CAGE‐C or CAGE +10 questions have higher sensitivity and specificity. Hence, more research is necessary to assess CAGE‐C and CAGE +10 questions.

Two points should be considered when interpreting the findings due to the differences between the included studies. There were six different reference standards or combinations used in the included studies (including the AUDIT, DSM‐III, DSM‐IV and ICD‐10) and 12 different descriptions of problematic alcohol use. Since 2013, with the introduction of the DSM‐5, the diagnoses of ‘alcohol abuse’ and ‘alcohol dependence’ have been combined into the diagnosis of AUD [4, 48, 49, 50]. Using one reliable reference standard (e.g., DSM‐5) and one description of problematic alcohol use (e.g., AUD) is recommended in future research to make comparisons easier. Furthermore, there were several specific populations (e.g., patients who were ≥65 years, adolescents and current drinkers). These populations often had extreme percentages of alcohol problems [27, 30, 33, 39], and this might influence the representativeness of the general adult population in hospitals.

This systematic review has some limitations and strengths. Starting with the limitations and strengths of the included studies. First, in some studies, extreme populations were tested (e.g., >30% of the population had alcohol problems), which may have influenced the DTA due to selection bias [51]. Second, ideally, one screening instrument should be compared with a reliable gold standard. However, this approach was lacking in some of the included studies (e.g., using the AUDIT as a reference standard), which might have led to differences in validity and reliability due to the similarities between the index test and the reference standard [33, 52]. Fourth, it is important to report the positive and negative predictive values in addition to the sensitivity and specificity, as they are dependent on the test itself and the prevalence (i.e., PPV = true positives/positive calls, NPV = true negative/positive calls) [21]. Unfortunately, these values were missing in the majority of the studies. Finally, performing the risk of bias assessment using the QUADAS‐2 tool showed that the greatest inconsistency was in blinding the results, which may have led to bias related to subjectivity [24]. Nevertheless, it is questionable whether it is even possible to blind the results if the reference standard is one of the screening instruments used for the patients [53]. Strengths of the included studies, despite, the lack of implementation of screening instruments for problematic alcohol use in hospitals in the Netherlands, the included studies show a variety of hospitals globally implementing and researching them.

In addition, our systemic review itself has limitations and strengths. First, although we only considered articles written in English, they were from all over the world. Second, the included studies represented the full adult population, including subpopulations in hospitals (patients in the ED, inpatients and outpatients). Third, in the full‐text inclusion phase studies, we decided to focus on the total outcomes in studies (i.e., not male and female outcomes separately) in order to compare the total outcomes with each other. Finally, although a meta‐analysis was not possible due to the heterogeneity among the included studies, we described screening instruments with the most accurate values (based on a sensitivity and specificity cutoff of >0.8) and provided an overview of all the variations and DTA within the studies to detect problematic alcohol use in hospitals among adults.

Our findings can be refined and utilised in future research, making them more comparable regarding the population, description of problematic alcohol use and the reference standard. Moreover, clinicians can now use this information and choose the cutoff they prefer when selecting a screening instrument for practical use, considering high sensitivity (i.e., includes ‘a problematic drinker’) and/or high specificity (i.e., excludes ‘not a problematic drinker’). Furthermore, the severity of problematic alcohol use clinicians aim to detect can differ. For example, the AUDIT with a cutoff of *≥*4 can be used to detect alcohol abuse and the AUDIT with a cutoff of ≥18 can be used to detect AUD.

## 5 CONCLUSION

When clinicians want to detect problematic alcohol use among patients, it is important to use the most accurate screening instruments. Based on our overview, clinicians in hospitals can now make an informed decision for inpatients and patients in the ED when choosing a screening instrument for detecting problematic alcohol use. The full AUDIT can be used for inpatients and patients in the ED. In addition, the AUDIT‐C and the RAPS4‐QF can be used for patients in the ED. More research is needed on outpatients and for screening instruments with high sensitivity and specificity that have only been validated with one study (i.e., the two‐question screener, DSM‐IV‐2, HOLD 5, CAGE‐C and CAGE +10 questions) in different hospital settings and with different patient populations.

## AUTHOR CONTRIBUTIONS

Conceptualisation (Jacqueline M. Bisschop, Hendrik J. M. de Jonge, Anja H. Brunsveld‐Reinders, Dike H. van de Mheen, Andrea D. Rozema); data curation (Jacqueline M. Bisschop, Hendrik J. M. de Jonge, Andrea D. Rozema); formal analysis (Jacqueline M. Bisschop, Hendrik J. M. de Jonge, Andrea D. Rozema); investigation (Jacqueline M. Bisschop); methodology (Jacqueline M. Bisschop, Hendrik J. M. de Jonge, Andrea D. Rozema); project administration (Jacqueline M. Bisschop); resources (Jacqueline M. Bisschop); software (Jacqueline M. Bisschop); supervision (Hendrik J. M. de Jonge, Anja H. Brunsveld‐Reinders, Dike H. van de Mheen, Andrea D. Rozema, Jolanda J. P. Mathijssen); validation (Jacqueline M. Bisschop, Hendrik J. M. de Jonge, Andrea D. Rozema); visualisation (Jacqueline M. Bisschop); writing—original draft (Jacqueline M. Bisschop); writing—review and editing (Jacqueline M. Bisschop, Hendrik J. M. de Jonge, Anja H. Brunsveld‐Reinders, Dike H. van de Mheen, Andrea D. Rozema, Jolanda J. P. Mathijssen). All authors have approved the final manuscript. Each author certifies that their contribution to this work meets the standards of the International Committee of Medical Journal Editors.

## CONFLICT OF INTEREST STATEMENT

All authors have no interest to declare.

## Appendix

**TABLE A1:** The search strategy for PubMed, PsycINFO and Embase.

Database	Search string	Number of articles
PubMed	#1 Search: (“Alcohol‐related disorders”[MeSH Terms] OR “alcohol misuse*”[Title/Abstract] OR “alcohol problem*”[Title/Abstract] OR “alcoholic intoxication*”[Title/Abstract] OR “drunkenness*”[Title/Abstract] OR “alcohol induced disorder*”[Title/Abstract] OR “alcohol induced disorder*”[Title/Abstract] OR “alcohol withdrawal*”[Title/Abstract] OR “alcohol related*”[Title/Abstract] OR “alcoholism*”[Title/Abstract] OR “alcohol dependen*”[Title/Abstract] OR “alcohol addiction*”[Title/Abstract] OR “alcohol use disorder*”[Title/Abstract] OR “alcohol abuse*”[Title/Abstract] OR “ethanol abuse*”[Title/Abstract] OR “binge drinking*”[Title/Abstract] OR “binge alcohol consumption*”[Title/Abstract] OR “problem drinking*”[Title/Abstract] OR “alcoholic individual*”[Title/Abstract] OR “alcoholics*”[Title/Abstract] OR “chronic alcohol*”[Title/Abstract] OR “dipsomania*”[Title/Abstract] OR “ethanol dependen*”[Title/Abstract])	155,839
	#2 Search: (“Surveys and Questionnaires”[MeSH Terms:noexp] OR “questionnaire*”[Title/Abstract] OR “survey*”[Title/Abstract] OR “respondent*”[Title/Abstract] OR “screening*”[Title/Abstract] OR “screeningsinstrument*”[Title/Abstract] OR “instrument*”[Title/Abstract] OR “scale*”[Title/Abstract] OR “assessment*”[Title/Abstract] OR “classification*”[Title/Abstract] OR “tool*”[Title/Abstract] OR “scoring*”[Title/Abstract]) AND (“Sensitivity and Specificity”[MeSH Terms] OR “Diagnostic Errors”[MeSH Terms] OR “sensitive”[Text Word] OR “sensitivity”[Text Word] OR “specificity”[Text Word] OR “accurate”[Text Word] OR “accuracy”[Text Word] OR “golden standard”[All Fields] OR “gold standard”[All Fields] OR (“reference”[Text Word] AND (“test”[Text Word] OR “standard”[Text Word])) OR “index test”[All Fields] OR “validity”[Text Word] OR “validation”[Text Word] OR “validate*”[Text Word] OR “valid”[Title] OR “verif*”[Title] OR “evaluation studies”[Publication Type] OR “evaluat*”[Title] OR (“false”[Text Word] AND (“positive”[Text Word] OR “negative”[Text Word])) OR “pretest”[Text Word] OR “pre‐test”[Text Word] OR “posttest”[Text Word] OR “post‐test”[Text Word] OR ((“predict”[All Fields] OR “predictabilities”[All Fields] OR “predictability”[All Fields] OR “predictable”[All Fields] OR “predictably”[All Fields] OR “predicted”[All Fields] OR “predicting”[All Fields] OR “prediction”[All Fields] OR “predictions”[All Fields] OR “predictive”[All Fields] OR “predictively”[All Fields] OR “predictiveness”[All Fields] OR “predictives”[All Fields] OR “predictivities”[All Fields] OR “predictivity”[All Fields] OR “predicts”[All Fields]) AND (“value”[All Fields] OR “values”[All Fields])) OR “predict*”[Title] OR “roc”[Text Word] OR “likelyhood”[Text Word] OR “likelihood”[Text Word] OR “value*”[Title] OR “reference values”[MeSH Terms] OR “cutoff”[Text Word] OR “cut‐off”[Text Word] OR “quality control”[MeSH Terms] OR “reproducibility of results”[MeSH Terms] OR “repeatability”[Text Word] OR “reproducibility”[Text Word] OR “efficacy”[Text Word] OR “reliability”[Text Word] OR “comparative study”[Publication Type] OR “odds”[Text Word] OR “error*”[Text Word] OR “suitability”[Text Word] OR “utility”[Text Word])	1,938,786
	#3 Search: “Hospitals”[Mesh] OR “Secondary Care Centers”[Mesh] OR “Secondary Care”[Mesh] OR “Hospital*”[tiab] OR “Emergency Unit*”[tiab] OR “Emergency Ward*”[tiab] OR “Emergency Department*”[tiab] OR “Emergency Room*”[tiab] OR “Emergency Outpatient Unit*”[tiab] OR “emergency cent*”[tiab] OR “Trauma center*”[tiab] OR “Trauma unit*”[tiab] OR “Medical cent*”[tiab] OR “Secondary Referral Center*”[tiab] OR “Secondary Care*”[tiab] OR “Secondary Care Center*”[tiab]	1,677,805
	#4 Search: #1 AND #2 AND #3 NOT ((“Adolescent”[MeSH Terms] OR “Child”[MeSH Terms] OR “Infant”[MeSH Terms] OR “adolescen*”[Title/Abstract] OR “child*”[Title/Abstract] OR “schoolchild*”[Title/Abstract] OR “infant*”[Title/Abstract] OR “girl*”[Title/Abstract] OR “boy”[Title/Abstract] OR “teen”[Title/Abstract] OR “teens”[Title/Abstract] OR “teenager*”[Title/Abstract] OR “youth*”[Title/Abstract] OR “pediatr*”[Title/Abstract] OR “paediatr*”[Title/Abstract] OR “puber*”[Title/Abstract]) NOT (“Adult”[MeSH Terms] OR “adult*”[Title/Abstract] OR “man”[Title/Abstract] OR “men”[Title/Abstract] OR “woman”[Title/Abstract] OR “women”[Title/Abstract]))	2182
	#5 Search: #1 AND #2 AND #3 NOT ((“Adolescent”[MeSH Terms] OR “Child”[MeSH Terms] OR “Infant”[MeSH Terms] OR “adolescen*”[Title/Abstract] OR “child*”[Title/Abstract] OR “schoolchild*”[Title/Abstract] OR “infant*”[Title/Abstract] OR “girl*”[Title/Abstract] OR “boy”[Title/Abstract] OR “teen”[Title/Abstract] OR “teens”[Title/Abstract] OR “teenager*”[Title/Abstract] OR “youth*”[Title/Abstract] OR “pediatr*”[Title/Abstract] OR “paediatr*”[Title/Abstract] OR “puber*”[Title/Abstract]) NOT (“Adult”[MeSH Terms] OR “adult*”[Title/Abstract] OR “man”[Title/Abstract] OR “men”[Title/Abstract] OR “woman”[Title/Abstract] OR “women”[Title/Abstract])) Filters: from 2001 to 2021	1621
PsycINFO	#1 exp. alcoholism/or (Alcohol misuse* or Alcohol Problem* or Alcoholic Intoxication* or Drunkenness* or Alcohol‐Induced Disorder* or Alcohol Induced Disorder* or Alcohol Withdrawal* or Alcohol Related* or Alcoholism* or Alcohol Dependen* or Alcohol Addiction* or AUD or Alcohol Abuse* or Ethanol Abuse* or Binge Drinking* or Binge Alcohol Consumption* or Problem drinking* or alcoholic individual* or alcoholics* or chronic alcohol* or dipsomania* or ethanol dependen*).ti,ab.	70,601
	#2 (exp psychometrics/or (sensitive or sensitivity or specificity or accurate or accuracy or golden standard or gold standard or index test or validity or validation or validate* or pretest or pre‐test or posttest or post‐test or predictive value or roc or likelihood or likelyhood or reproducibility or efficacy or reliability or repeatability or cutoff or cut‐off or odds or error* or suitability or utility).mp. or (reference and (test or standard)).mp. or (false and (positive or negative)).mp. or (valid or verif* or evaluat* or predict* or value*).ti,ab.) and (exp Questionnaires/or (Questionnaire* or Survey* or Respondent* or Screening* or screeningsinstrument* or instrument* or scale* or assessment* or classification* or tool* or scoring*).ti,ab.)	744,680
	#3 exp. hospitals/or exp. hospitalised Patients/or exp. Emergency Services/or exp. Emergency Medicine/or (Hospital* or Emergency Unit* or Emergency Ward* or Emergency Department* or Emergency Room* or Emergency Outpatient Unit* or Emergency cent* or Trauma center* or Trauma unit* or Medical cent* or Secondary Referral Center* or Secondary Care* or Secondary Care Center*).ti,ab.	192,208
	#4 (1 and 2 and 3) not (((childhood <birth to 12 years> or adolescence <13 to 17 years>).ag. or (adolescen* or child* or schoolchild* or infant* or girl* or boy* or teen or teens or teenager* or youth* or pediatr* or paediatr* or puber*).ti,ab.) not (adulthood <18+ years>.ag. or (adult* or man or men or woman or women).ti,ab.))	1178
	#5 4	1178
	#6 limit 5 to yr. = “2001–Current”	764
Embase	#1 exp. Alcoholism/or (Alcohol misuse* or Alcohol Problem* or Alcoholic Intoxication* or Drunkenness* or Alcohol‐Induced Disorder* or Alcohol Induced Disorder* or Alcohol Withdrawal* or Alcohol Related* or Alcoholism* or Alcohol Dependen* or Alcohol Addiction* or AUD* or Alcohol Abuse* or Ethanol Abuse* or Binge Drinking* or Binge Alcohol Consumption* or Problem drinking* or alcoholic individual* or alcoholics* or chronic alcohol* or dipsomania* or ethanol dependen*).ti,ab.	481,962
	#2 (exp “sensitivity and specificity”/or exp. diagnostic error/or exp. reference value/or exp. quality control/or exp. measurement precision/or (sensitive or sensitivity or specificity or accurate or accuracy or golden standard or gold standard or index test or validity or validation or validate* or pretest or pre‐test or posttest or post‐test or predictive value or roc or likelihood or likelyhood or reproducibility or efficacy or reliability or repeatability or cutoff or cut‐off or odds or error* or suitability or utility).mp. or (reference and (test or standard)).mp. or (false and (positive or negative)).mp. or (valid or verif* or evaluat* or predict* or value*).ti.) and (exp Questionnaire/or (Questionnaire* or Survey* or Respondent* or Screening* or screeningsinstrument* or instrument* or scale* or assessment* or classification* or tool* or scoring*).ti,ab.)	2,450,250
	#3 exp. Secondary health care/or exp. Hospital/or exp. emergency ward/or (Hospital* or Emergency Unit* or Emergency Ward* or Emergency Department* or Emergency Room* or Emergency Outpatient Unit* or Emergency cent* or Trauma center* or Trauma unit* or Medical cent* or Secondary Referral Center* or Secondary Care* or Secondary Care Center*).ti,ab.	2,841,280
	#4 (1 and 2 and 3) not ((exp adolescent/or exp. child/or (adolescent* or child* or schoolchild* or infant* or girl* or boy* or teen* or teenager* or youth* or pediatr* or paediatr* or puber*).ti,ab,kw.) not (exp ‘adult’/or exp. ‘aged’/or exp. ‘middle aged’/or (adult* or man or men or woman or women).ti,ab,kw.))	13,234
	#5 4 not (conference review or conference paper or conference abstract or books or chapter).pt.	6544
	#6 5	6544
	#7 limit 6 to yr. = “2001–Current”	5603

**TABLE A2:** The complete list of the diagnostic test accuracy of the included studies.

Author/s (year) [reference]	Screening instruments	Sensitivity (95% confidence interval)	Specificity (95% confidence interval)	Positive predictive value	Negative predictive value
Shenvi et al. (2020) [27]	2‐Question	**0.98 (0.93–1.0)**	**0.87 (0.80–0.92)**		
	AUDIT >4	0.93 (0.85–0.97)	0.78 (0.70–0.84)		
	AUDIT >8	0.52 (0.41–0.63)	0.99 (0.95–1.0)		
	CAGE	0.45 (0.34–0.56)	1.0 (0.97–1.0)		
Larsson and Nehlin (2016) [28]	AUDIT‐C	**F 0.86, M 0.84, T 0.85**	**F 0.92, M 0.83, T 0.88**		
	AUDIT‐3	F 0.41, M 0.68, T 0.55	F 0.99, M 0.93, T 0.96		
	WCQ	F 0.14, M 0.09, T 0.12	F 1.0, M 1.0, T 1.0		
	HED	F 0.63, M 0.74, T 0.69	F 0.94, M 0.89, T 0.91		
Fulbrook et al. (2015) [29]	Australian PAT cutoff of 8	M 0.76 (0.71–0.81)	M 0.85 (0.81–0.89)		
		F 0.64 (0.59–0.70)	F 0.95 (0.93–0.98)		
		Age ≤25, 0.80 (72.7–88.1)	0.79 (0.71–0.87)		
		Age >25, 0.69 (65.0–72.8)	0.92 (0.90–0.95)		
		**T 0.73** (0.69–0.76)	**T 0.91** (88.6–93.0)		
Geneste et al. (2012) [30]	RAPS4	RAPS4	RAPS4		
	RAPS4‐QF	Abuse/harmful drinking/dependence	Abuse/harmful drinking/dependence		
	CAGE	**≥1 T 095, M 0.96**, F 0.94	≥1 T **0.65, M 0.9**, F 0.29		
	AUDIT	≥2 T 0.8, M 0.76, **F 0.91**	≥2 T 0.82, M 1.0, **F 0.57**		
		Dependence	Dependence		
		≥1 T 0.96, **M 0.96**, F 0.97	≥1 T 0.36, **M 0.42**, F 0.25		
		≥ 2 T **0.84**, M 0.79, **F 0.97**	≥ 2 T **0.64**, M 0.71, **F 0.5**		
		RAPS4‐QF	RAPS4‐QF		
		Abuse/harmful drinking/dependence	Abuse/harmful drinking/dependence		
		≥2 T 0.99, **M 1.0**, F 0.97	≥2 T 0.35, **M 0.6**, F 0.0		
		**≥3 T 0.89**, M 0.88, F 0.91	≥3 **T 0.77**, M 1.0, F 0.43		
		≥4 T 0.73, M 0.69, **F 0.89**	≥4 T 0.88, M 1.0, **F 0.71**		
		Dependence	Dependence		
		≥3 **T 0.92, M 0.91**, F 0.97	≥3 **T 0.53, M 0.58**, F 0.42		
		≥4 T 0.77, M 0.72, **F 0.93**	≥4 T 0.69, M 0.75, **F 0.58**		
		CAGE	CAGE		
		Abuse/harmful drinking/dependence	Abuse/harmful drinking/dependence		
		≥2 **T 0.94, M 0.92**, F 1.0	≥2 **T 0.65, M 0.6**, F 0.71		
		≥3 T 0.84, M 0.84, **F 0.86**	≥3 T 0.76, M 0.8, **F 0.71**		
		Dependence	Dependence		
		≥ 2 T 0.96, M 0.95, F 1.0	≥ 2 T 0.42, M 0.41, F 0.42		
		**≥ 3 T 0.89, M 0.88, F 0.93**	**≥ 3 T 0.61, M 0.58, F 0.67**		
		AUDIT	AUDIT		
		Abuse/harmful drinking/dependence	Abuse/harmful drinking/dependence		
		≥7 T 0.99, M 0.98, **F 1.0**	≥7 T 0.53, M 0.4, **F 0.71**		
		≥8 T 0.94, M 0.94, F 0.94	≥8 T 0.58, M 0.5, F 0.71		
		**≥ 12 T 0.88, M 0.88**, F 0.89	≥ **12 T 0.88, M 1.0**, F 0.71		
		Dependence	Dependence		
		≥8 T 0.97, M 0.96, F 1.0	≥8 T 0.42, M 0.33, F 0.58		
			≥11 T 0.53, M 0.5, **F 0.58**		
		≥11 T 0.92, M 0.9, **F 1.0**	≥14 T 0.69, **M 0.71**, F 0.67		
		≥14 T 0.87, **M 0.86**, F 0.9	≥**18 T 0.83**, M 0.79, F 0.92		
		≥18 **T 0.8**, M 0.81, F 0.8			
Richoux et al. (2011) [31]	CAGE	**0.75**	**0.92**		
	AUDIT	**0.87**	**0.80**		
Okay et al. (2010) [32]	MAST	**0.74**	**0.98**		
Kelly et al. (2009) [33]	AUDIT‐C	AUDIT‐C	AUDIT‐C		
	FAST	3 T 0.97, M 0.96, F 1.0	3 T 0.32, M 0.23, F 0.40		
	RAPS4‐QF	4 T 0.94, M 0.94, F 0.94	4 T 0.47, M 0.36, F 0.57		
	RUFT‐Cut	5 T 0.82, M 0.83, **F 0.77**	5 T 0.67, M 0.55, **F 0.78**		
	CRAFFT	**6 T 0.74, M 0.77**, F 0.65	**6 T 0.77, M 0.68**, F 0.85		
	DSM‐IV‐2	7 T 0.60, M 0.65, F 0.47	7 T 0.89, M 0.86, F 0.92		
		FAST	FAST		
		2 T 0.89, M 0.88, F 0.94	2 T 0.53, M 0.39, F 0.65		
		**3 T 0.83, M 0.79, F 0.94**	**3 T 0.73, M 0.68, F 0.78**		
		4 T 0.75, M 0.73, F 0.82	4 T 0.82, M 0.75, F 0.88		
		5 T 0.59, M 0.54, F 0.71	5 T 0.89, M 0.86, F 0.92		
		RAPS4‐QF	RAPS4‐QF		
		1 T 1.0, M 1.0, F 1.0	1 T = 0.25, M = 0.14, F = 0.30		
		2 T 0.87, M 0.85, F 0.94	2 T = 0.51, M = 0.43, F = 0.58		
		**3 T 0.79, M 0.79, F 0.77**	**3 T = 0.72, M = 0.66, F = 0.77**		
		4 T 0.55, M 0.54, F 0.59	4 T = 0.86, M = 0.82, F = 0.90		
		RUFT‐Cut	RUFT‐Cut		
		1 T 0.94, M 92, F 1.0	1 T 0.21, M 0.12, F 0.28		
		2 T 0.89, M 0.88, F 0.94	2 T 0.43, M 0.37, F 0.48		
		**3 T 0.80, M 0.77, F 0.88**	**3 T 0.68, M 0.68, F 0.68**		
		4 T 0.69, M 0.69, F 0.71	4 T 0.83, M 0.79, F 0.87		
		CRAFFT	CRAFFT		
		1 T 0.97, M 0.96, F 1.0	1 T 0.22, M 0.16, F 0.28		
		2 T 0.94, M 0.94, F 0.94	2 T 0.49, M 0.44, F 0.52		
		**3 T 0.69, M 0.71, F 0.65**	**3 T 0.73, M 0.68, F 0.78**		
		4 T 0.51, M 0.52, F 0.47	4 T 0.85, M 0.82, F 0.88		
		DSM‐IV‐2	DSM‐IV‐2		
		**1 T 0.88, M 0.85, F 0.94**	**1 T 0.90, M 87, F 0.92**		
		2 T 0.26, M 0.25, F 0.29	2 T 0.98, M 0.82, F 0.98		
Cremonte and Cherpitel (2008) [34]	AUDIT	AUDIT	AUDIT		
	CAGE	Abuse DSM 0.65	0.77		
	Brief MAST	Harmful ICD 0.68	0.77		
	RAPS4‐QF	Depend. DSM/ICD 0.93	0.80		
	TWEAK				
		CAGE	CAGE		
		Abuse DSM 0.48	0.82		
		Harmful ICD 0.51	0.82		
		Depend. DSM/ICD 0.83	0.86		
		Brief MAST	Brief MAST		
		Abuse DSM 0.04	0.95		
		Harmful ICD 0.04	0.95		
		Depend. DSM/ICD 0.44	0.99		
		RAPS4 Q‐F	RAPS4‐QF		
		Abuse DSM 0.86	0.64		
		Harmful ICD 0.88	0.68		
		Depend. DSM/ICD 0.89	0.87		
		TWEAK	TWEAK		
		Abuse DSM 0.82	0.65		
		Harmful ICD 0.66	0.62		
		Depend. DSM/ICD 0.98	0.67		
Bradshaw et al. (2008) [35]	FAST	Above guideline 0.89	0.94		
		Alcohol abuse 1.0	0.73		
Zierau et al. (2005) [36]	CAGE‐C (Copenhagen) modified CAGE test (“Ever” has been replaced to “last year” and there are two additional questions)	0.94 (0.82–0.99)	0.88 (0.83–0.89)	0.73 (0.63–0.77)	0.98 (0.93–0.99)
Chen et al. (2005) [37]	AUDIT (Chinese version)	AUDIT 7: 1.00	0.86 (0.83–0.89)	0.59 (0.49–0.68)	1.00
		AUDIT 8**: 0.97 (0.87–0.99)**	**0.90 (0.87–0.92)**	**0.66 (0.55–0.75)**	**0.99 (0.97–1.00)**
		AUDIT 9: 0.90 (0.79–0.95)	0.92 (0.89–0.94)	0.69 (0.58–0.78)	0.98 (0.95–0.99)
		AUDIT 10:0.88 (0.77–0.94)	0.94 (0.91–0.96)	0.73 (0.62–0.82)	0.98 (0.95–0.99)
		AUDIT 11: 0.83 (0.71–0.91)	0.95 (0.93–0.97)	0.78 (0.66–0.86)	0.97 (0.94–0.98)
Castells and Furlanetto (2005) [38]	CAGE	**CAGE 1: 0.94**	**0.86**	**0.31**	**1.0**
	+ 10 added questions	CAGE 2: 0.83	0.92	0.42	0.99
	Brazilian version	CAGE 3: 0.54	0.97	0.55	0.97
		CAGE 4: 0.13	1.0	0.67	0.94
Kelly et al. (2004) [39]	AUDIT	AUDIT	AUDIT	AUDIT	AUDIT
	CAGE	3 0.97	0.18	0.43	0.88
	CRAFFT	4 0.94	0.22	0.44	0.82
	RAPS4‐QF	5 0.90	0.34	0.49	0.85
		6 0.89	0.49	0.52	0.87
		7 0.87	0.54	0.55	0.86
		8 0.87	0.65	0.60	0.88
		9 0.84	0.73	0.66	0.86
		**10 0.82**	**0.78**	**0.71**	**0.88**
		11 0.74	0.80	0.72	0.84
		12 0.71	0.84	0.74	0.84
		CAGE	CAGE	CAGE	CAGE
		**1 0.66**	**0.58**	**0.52**	**0.71**
		2 0.53	0.78	0.63	0.70
		3 0.21	0.82	0.89	0.64
		CRAFFT	CRAFFT	CRAFFT	CRAFFT
		1 0.95	0.20	0.45	0.85
		2 0.95	0.44	0.54	0.92
		**3 0.82**	**0.67**	**0.63**	**0.84**
		4 0.61	0.80	0.68	0.75
		5 0.29	0.93	0.73	0.65
		RAPS‐QF	RAPS‐QF	RAPS‐QF	RAPS‐QF
		1 0.97	0.09	0.42	0.83
		2 0.90	0.29	0.47	0.80
		**3 0.82**	**0.54**	**0.55**	**0.81**
		4 0.68	0.80	0.70	0.79
		5 0.34	0.93	0.76	0.67
Cherpitel and Bazargan (2003) [40]	AUDIT	AUDIT	AUDIT		
	RAPS4	Alcohol dependence	Alcohol dependence		
	RAPS4‐QF	T 0.87, M 0.84, F 1.0	T 0.92, M 0.87, F 0.97		
		African American 0.86	African American 0.91		
		Hispanic 0.87	Hispanic 0.92		
			Alcohol Abuse		
			T 0.95, M 0.91, F 0.98		
			African American 0.94		
			Hispanic 0.96		
			RAPS4		
			Alcohol dependence		
			T 0.90, M 0.84, F 0.97		
			African		
			American 0. 86		
			Hispanic 0.93		
			Alcohol Abuse		
			T 0.93, M 0.89, F 0.97		
			African American0. 89		
			Hispanic 0.96		
			RAPS4‐QF		
			Alcohol Abuse T 0.83, M 0.73, F 0.93		
			African American 0.75 Hispanic 0.89		
Hodgson et al. (2002) [41]	FAST	Fracture clinic 0.94	Fracture clinic 0.89		
		Primary care 0.91	Primary care 0. 95		
		Dental hospital 0.97	Dental hospital 0.91		
		ED 0.94	ED 0.86		
Hearne et al. (2002) [15]	CAGE	CAGE ≥2 0.77	0.99	0.94	0.95
	AUDIT	AUDIT >8 0.89	0.91	0.70	0.90
	BMAST	AUDIT >10 0.75	0.97	0.84	0.93
		AUDIT >12 0.60	0.98	0.88	0.91
		BMAST ≥5 0.37	1.0	1.0	0.88
Aertgeerts et al. (2002) [42]	CAGE	CAGE ≥1 **0.72**	**0.85**	0.41	0.96
	AUDIT	CAGE ≥2 0.41	0.95	0.52	0.92
	AUDIT‐C	**AUDIT ≥5 0.83**	**0.85**	**0.44**	**0.97**
	AUDIT‐PC	AUDIT ≥6 0.72	0.90	0.51	0.96
	Fiveshot	AUDIT ≥7 0.69	0.93	0.57	0.96
		AUDIT ≥8 0.66	0.96	0.68	0.95
		AUDIT‐C ≥ **5 0.69**	**0.87**	0.43	0.95
		AUDIT‐C ≥ 6 0.66	0.94	0.59	0.95
		AUDIT‐C ≥ 7 0.62	0.95	0.64	0.95
		AUDIT‐C ≥ 8 0.52	0.97	0.71	0.93
		AUDIT‐PC ≥5 **0.69**	**0.91**	0.53	0.95
		AUDIT‐PC ≥6 0.59	0.96	0.65	0.94
		AUDIT‐PC ≥7 0.55	0.98	0.80	0.94
		AUDIT‐PC ≥8 0.41	0.98	0.75	0.92
		FIVESHOT ≥1.5 0.90	0.66	0.27	0.98
		FIVESHOT ≥2.0 0.86	0.79	0.37	0.98
		**FIVESHOT ≥2.5 0.79**	**0.88**	**0.48**	**0.97**
		FIVESHOT ≥3.0 0.59	0.93	0.55	0.94
Gomez et al. (2001) [43]	CAGE	0.70 (0.60–078)	0.75 (0.64–0.84)	0.78	0.67
Cherpitel (2001) [44]	CAGE	CAGE PC 0.82 ED 0.89	CAGE PC 0.98 ED 0.94		
	BMAST	BMAST PC 0.33 ED 0.28	BMAST PC 0.99 ED 0.99		
	AUDIT	AUDIT PC 0.67 ED 0.93	AUDIT PC 0.97 ED 0.94		
	TWEAK	TWEAK PC 0.75 ED 0.89	TWEAK PC 0.97 ED 0.92		
	RAPS Two tolerance questions: hold at least five drinks (HOLD 5) and drink ≥5 drinks monthly (5 monthly)	RAPS PC 0.83 ED 0.97	RAPS PC 0.97 ED 0.86		
		HOLD (≥ 5) PC 0.55 ED 0.83 5 MONTHLY PC 0.50 ED 0.74	HOLD (≥ 5) PC 0.93 ED 0.82 5 MONTHLY PC 0.96 ED 0.92		
		Alcohol Abuse T 0.79, M 0.77, F 0.93 African American 0.79 Hispanic 0.79 RAPS4 Alcohol dependence T 0.89, M 0.87, F 1.0 African American 0.93 Hispanic 84 Alcohol Abuse T 0.82, M 0.80, F 0.93 African American 0.87 Hispanic 0.77 RAPS4‐QF Alcohol Abuse T 0.98, M 0.98, F 1.0 African American 0.96 Hispanic 1.0			
Borges and Cherpitel (2001) [45]	RAPS	RAPS for ADS 0.93	0.79		
		RAPS for ADAAHM 0.74	0.85		

Abbreviations: AUDIT, Alcohol Use Disorder Identification Test; BMAST, Brief Michigan Alcohol Screening Test; CAGE, Cutting down, Annoyance by criticism, Guilty feeling, Eye‐openers; CRAFFT, Car, Relax, Alone, Forget, Friends, Trouble; DSM, Diagnostic and Statistical Manual of Mental Disorders; FAST, Functional Analysis Screening Tool; HED, heavy episodic drinking; ICD‐10, International Statistical Classification of Diseases and Related Health Problems 10th Revision; MAST, Michigan Alcohol Screening Test; PAT, Paddington Alcohol Test; RAPS‐4, Rapid Alcohol Problems Screen 4 questions; RAPS‐QF, Rapid Alcohol Problems Screen 4 + quantity and frequency question; TWEAK, Tolerance, Worried, Eye opener, Amnesia, Cut down; WCQ, weekly consumption question.

## References

[1] Rehm J , Manthey J , Shield KD , Ferreira‐Borges C . Trends in substance use and in the attributable burden of disease and mortality in the WHO European Region, 2010–16. Eur. J. Public Health. 2019;29(4):723–728. 10.1093/eurpub/ckz064 31008515

[2] World Health Organization . Global status report on alcohol and health 2018. 2019.

[3] Bryazka D , Reitsma MB , Griswold MG , Abate KH , Abbafati C , Abbasi‐Kangevari M , et al. Population‐level risks of alcohol consumption by amount, geography, age, sex, and year: a systematic analysis for the Global Burden of Disease Study 2020. The Lancet. 2022;400(10347):185–235. 10.1016/S0140-6736(22)00847-9 PMC9289789 35843246

[4] APA . Diagnostic and statistical manual of mental disorders: DSM‐5. 5th ed. Washington, D.C.: American Psychiatric Pub; 2013.

[5] Chen C‐H , Chen WJ , Cheng AT . Prevalence and identification of alcohol use disorders among nonpsychiatric inpatients in one general hospital. Gen. Hosp. Psychiatry. 2004;26(3):219–225. 10.1016/j.genhosppsych.2004.01.001 15121350

[6] Wakeman SE , Herman G , Wilens TE , Regan S . The prevalence of unhealthy alcohol and drug use among inpatients in a general hospital. Subst. Abuse. 2020;41(3):331–339. 10.1080/08897077.2019.1635961 31368860

[7] Grønbaek M . The positive and negative health effects of alcohol‐and the public health implications. J. Intern. Med. 2009;265(4):407–420. 10.1111/j.1365-2796.2009.02082.x 19298457

[8] Scarborough H , Piercey, W. Douglas and Fralick, Pamela C. "hospital": Encyclopedia Britannica. 2023. [Accessed 30 June 2023]. Available from: https://www.britannica.com/science/hospital.

[9] Barata IA , Shandro JR , Montgomery M , Polansky R , Sachs CJ , Duber HC , et al. Effectiveness of SBIRT for alcohol use disorders in the emergency department: a systematic review. West. J. Emerg. Med. 2017;18(6):1143. 10.5811/westjem.2017.7.34373 29085549 PMC5654886

[10] Barbosa C , Cowell A , Dowd W , Landwehr J , Aldridge A , Bray J . The cost‐effectiveness of brief intervention versus brief treatment of screening, brief intervention and referral to treatment (SBIRT) in the United States. Addiction. 2017;112:73–81. 10.1111/add.13658 28074567

[11] Babor TF , Del Boca F , Bray JW . Screening, brief intervention and referral to treatment: implications of SAMHSA's SBIRT initiative for substance abuse policy and practice. Addiction. 2017;112:110–117. 10.1111/add.13675 28074569

[12] Kools N , van de Goor I , Bovens R , van de Mheen D , Rozema AD . Impeding and facilitating factors for the implementation of alcohol interventions in hospitals: a qualitative and exploratory study among Dutch healthcare professionals. BMC Health Serv. Res. 2022;22(1):6. 10.1186/s12913-021-07412-1 34974830 PMC8722137

[13] Hydes T , Gilmore W , Sheron N , Gilmore I . Treating alcohol‐related liver disease from a public health perspective. J. Hepatol. 2019;70(2):223–236. 10.1016/j.jhep.2018.10 30658724

[14] Cohen E , Feinn R , Arias A , Kranzler HR . Alcohol treatment utilization: findings from the national epidemiologic survey on alcohol and related conditions. Drug and alcohol depend. 2007;86(2‐3):214–221. 10.1016/j.drugalcdep.2006.06.008 16919401

[15] Hearne R , Connolly A , Sheehan J . Alcohol abuse: prevalence and detection in a general hospital. J. R. Soc. Med. 2002;95(2):84–87. 10.1258/jrsm.95.2.84 11823551 PMC1279317

[16] Draper B , Ridley N , Johnco C , Withall A , Sim W , Freeman M , et al. Screening for alcohol and substance use for older people in geriatric hospital and community health settings. Int. Psychogeriatr. 2015;27(1):157–166. 10.1017/s1041610214002014 25247846

[17] Verhalle L , Van Bockstaele K , Duerinckx N , Vanhoof J , Dierickx K , Neyens L , et al. How to screen for at‐risk alcohol use in transplant patients? From instrument selection to implementation of the AUDIT‐C. Clin. Transplant. 2021;35(1):e14137. 10.1111/ctr.14137 33141977

[18] Jones LA . Systematic review of alcohol screening tools for use in the emergency department. Emerg. Med. J. 2011;28(3):182–191. 10.1136/emj.2009.085324 20947921

[19] PROSPERO . 2022. [Accessed January 2022] Available from: https://www.crd.york.ac.uk/prospero/display_record.php?ID=CRD42022291306

[20] Page MJ , McKenzie JE , Bossuyt PM , Boutron I , Hoffmann TC , Mulrow CD , et al. The PRISMA 2020 statement: an updated guideline for reporting systematic reviews. BMJ. 2021;372:n71. 10.1136/bmj.n71 33782057 PMC8005924

[21] Macaskill P , Gatsonis C , Deeks J , Harbord R , Takwoingi Y . Cochrane handbook for systematic reviews of diagnostic test accuracy, version 0.9. London: The Cochrane Collaboration; 2010.

[22] Innovation VH . Covidence systematic review software Melbourne, Australia 2022. [Accessed February 2022] Available from: www.covidence.org.

[23] Ouzzani M , Hammady H , Fedorowicz Z , Elmagarmid A . Rayyan‐a web and mobile app for systematic reviews. Syst. Rev. 2016;5(1):210. 10.1186/s13643-016-0384-4 27919275 PMC5139140

[24] Whiting PF , Rutjes AW , Westwood ME , Mallett S , Deeks JJ , Reitsma JB , et al. QUADAS‐2: a revised tool for the quality assessment of diagnostic accuracy studies. Ann. Intern. Med. 2011;155(8):529–536. 10.7326/0003-4819-155-8-201110180-00009 22007046

[25] Campbell M , McKenzie JE , Sowden A , Katikireddi SV , Brennan SE , Ellis S , et al. Synthesis without meta‐analysis (SWiM) in systematic reviews: reporting guideline. BMJ. 2020;368:l6890. 10.1136/bmj.l6890 31948937 PMC7190266

[26] Bramer WM , Giustini D , de Jonge GB , Holland L , Bekhuis T . De‐duplication of database search results for systematic reviews in EndNote. J. Med. Libr. Assoc. 2016;104(3):240–243. 10.3163/1536-5050.104.3.014 27366130 PMC4915647

[27] Shenvi CL , Weaver MA , Biese KJ , Wang Y , Revankar R , Fatade Y , et al. Identification and characterization of older emergency department patients with high‐risk alcohol use. J Am Coll Emerg Physicians Open. 2020;1(5):804–811. 10.1002/emp2.12196 33145524 PMC7593462

[28] Larsson K , Nehlin C . Screening accuracy of brief alcohol screening instruments in a general hospital setting. Scand. J. Public Health. 2016;44(6):599–603. 10.1177/1403494816651779 27236143

[29] Fulbrook P , Lawrence P , Watt K . Validity of the paddington alcohol test in an Australian emergency department. Alcohol Alcohol. 2015;50(4):407–412. 10.1093/alcalc/agv024 25795732

[30] Geneste J , Pereira B , Arnaud B , Christol N , Liotier J , Blanc O , et al. CAGE, RAPS4, RAPS4‐QF and AUDIT screening tests for men and women admitted for acute alcohol intoxication to an emergency department: are standard thresholds appropriate? Alcohol Alcohol. 2012;47(3):273–281. 10.1093/alcalc/ags027 22414922 PMC3331621

[31] Richoux C , Ferrand I , Casalino E , Fleury B , Ginsburg C , Lejoyeux M . Alcohol use disorders in the emergency ward: choice of the best mode of assessment and identification of at‐risk situations. Int. J. Emerg. Med. 2011;4(1):27. 10.1186/1865-1380-4-27 21672196 PMC3125196

[32] Okay T , Sengul C , Acikgoz C , Ozan G , Dilbaz N . Prevalence of alcohol dependence and abuse in a general hospital; Sensitivity and specificity of Mast. Eur. J. Gen. Med. 2010;7(1):9–16. 10.29333/ejgm/82787

[33] Kelly TM , Donovan JE , Chung T , Bukstein OG , Cornelius JR . Brief screens for detecting alcohol use disorder among 18‐20 year old young adults in emergency departments: comparing AUDIT‐C, CRAFFT, RAPS4‐QF, FAST, RUFT‐Cut, and DSM‐IV 2‐Item Scale. Addict. Behav. 2009;34(8):668–674. 10.1016/j.addbeh.2009.0 19398161 PMC3217267

[34] Cremonte M , Cherpitel CJ . Performance of screening instruments for alcohol use disorders in emergency department patients in Argentina. Substance Use and Misuse. 2008;43(1):125–138. 10.1080/10826080701212337 18189209

[35] Bradshaw P , Denny M , Cassidy EM . Alcohol misuse in the general hospital: some hard facts. Ir. J. Med. Sci. 2008;177(4):339–342. 10.1007/s11845-008-0243-y 18953625

[36] Zierau F , Hardt F , Henriksen JH , Holm SS , Jørring S , Melsen T , et al. Validation of a self‐administered modified CAGE test (CAGE‐C) in a somatic hospital ward: comparison with biochemical markers. Scand. J. Clin. Lab. Invest. 2005;65(7):615–622. 10.1080/00365510500333445 16271993

[37] Chen C‐H , Chen WJ , Cheng ATA . New approach to the validity of the alcohol use disorders identification test: stratum‐specific likelihood ratios analysis. Alcohol. Clin. Exp. Res. 2005;29(4):602–608. 10.1097/01.alc.0000159189.566 15834225

[38] Castells MA , Furlanetto LM . Validity of the CAGE questionnaire for screening alcohol‐dependent inpatients on hospital wards. Braz J Psychiatry. 2005;27(1):54–57. 10.1590/s1516-44462005000100012 15867984

[39] Kelly TM , Donovan JE , Chung T , Cook RL , Delbridge TR . Alcohol use disorders among emergency department‐treated older adolescents: a new brief screen (RUFT‐Cut) using the AUDIT, CAGE, CRAFFT, and RAPS‐QF. Alcohol. Clin. Exp. Res. 2004;28(5):746–753. 10.1097/01.alc.0000125346.37075.85 15166649

[40] Cherpitel CJ , Bazargan S . Screening for alcohol problems: comparison of the audit, RAPS4 and RAPS4‐QF among African American and Hispanic patients in an inner city emergency department. Drug Alcohol Depend. 2003;71(3):275–280. 10.1016/s0376-8716(03)00140-6 12957345

[41] Hodgson R , Alwyn T , John B , Thom B , Smith A . The fast alcohol screening test. Alcohol Alcohol. 2002;37(1):61–66. 10.1093/alcalc/37.1.61 11825859

[42] Aertgeerts B , Buntinx F , Ansoms S , Fevery J . Questionnaires are better than laboratory tests to screen for current alcohol abuse or dependence in a male inpatient population. Acta Clin. Belg. 2002;57(5):241–249. 10.1179/acb.2002.048 12534130

[43] Gomez A , Conde A , Aguiar JA , Santana JM , Jorrin A , Betancor P . Diagnostic usefulness of carbohydrate‐deficient transferrin for detecting alcohol‐related problems in hospitalized patients. Alcohol Alcohol. 2001;36(3):266–270. 10.1093/alcalc/36.3.266 11373266

[44] Cherpitel CJ . Screening for alcohol problems: a comparison of instrument performance among black emergency department and primary care patients. J. Subst. Abus. 2001;5(4):290–297. 10.3109/14659890109059826

[45] Borges G , Cherpitel CJ . Selection of screening items for alcohol abuse and alcohol dependence among Mexicans and Mexican Americans in the emergency department. J. Stud. Alcohol. 2001;62(3):277–285. 10.15288/jsa.2001.62.277 11414336

[46] Joyce J. Bayes' theorem The (Stanford) encyclopedia of philosophy: Edward N. Zalta; 2021[Accessed May 2022].

[47] de Meneses‐Gaya C , Zuardi AW , Loureiro SR , Crippa JAS . Alcohol use disorders identification test (AUDIT): an updated systematic review of psychometric properties. Psychol Neurosci. 2009;2(1):83–97. 10.3922/j.psns.2009.1.12

[48] Goldstein RB , Chou SP , Smith SM , Jung J , Zhang H , Saha TD , et al. Nosologic comparisons of DSM‐IV and DSM‐5 alcohol and drug use disorders: results from the national epidemiologic survey on alcohol and related conditions‐III. J. Stud. Alcohol Drugs. 2015;76(3):378–388. 10.15288/jsad.2015.76.378 25978823 PMC4440296

[49] Bartoli F , Carrà G , Crocamo C , Clerici M . From DSM‐IV to DSM‐5 alcohol use disorder: an overview of epidemiological data. Addict. Behav. 2015;41:46–50. 10.1016/j.addbeh.2014.09.029 25305657

[50] Lago L , Bruno R , Degenhardt L . Concordance of ICD‐11 and DSM‐5 definitions of alcohol and cannabis use disorders: a population survey. Lancet Psychiatry. 2016;3(7):673–684. 10.1016/s2215-0366(16)00088-2 27371989

[51] Delgado‐Rodríguez M , Llorca J . Bias. J. Epidemiol. Community Health. 2004;58(8):635–641. 10.1136/jech.2003.008466 15252064 PMC1732856

[52] Streiner DL , Norman GR , Cairney J . Health measurement scales: a practical guide to their development and use. 5th ed. New York, NY, US: Oxford University Press; 2015.

[53] Shafer SL , Dexter F . Publication bias, retrospective bias, and reproducibility of significant results in observational studies. Anesthesia & Analgesia. 2012;114(5):931–932. 10.1213/ANE.0b013e31824a0b5b 22523409

